# Catalytic Depolymerization of Waste Polyolefins by Induction Heating: Selective Alkane/Alkene Production

**DOI:** 10.1021/acs.iecr.1c02674

**Published:** 2021-10-14

**Authors:** Bernard Whajah, Natalia da Silva Moura, Justin Blanchard, Scott Wicker, Karleigh Gandar, James A. Dorman, Kerry M. Dooley

**Affiliations:** † Cain Department of Chemical Engineering, Louisiana State University, Baton Rouge, Louisiana 70803, United States; ‡ Department of Chemistry, Rhodes College, Memphis, Tennessee 38112, United States; § Science Department, Baton Rouge Community College, Baton Rouge, Louisiana 70806, United States

## Abstract

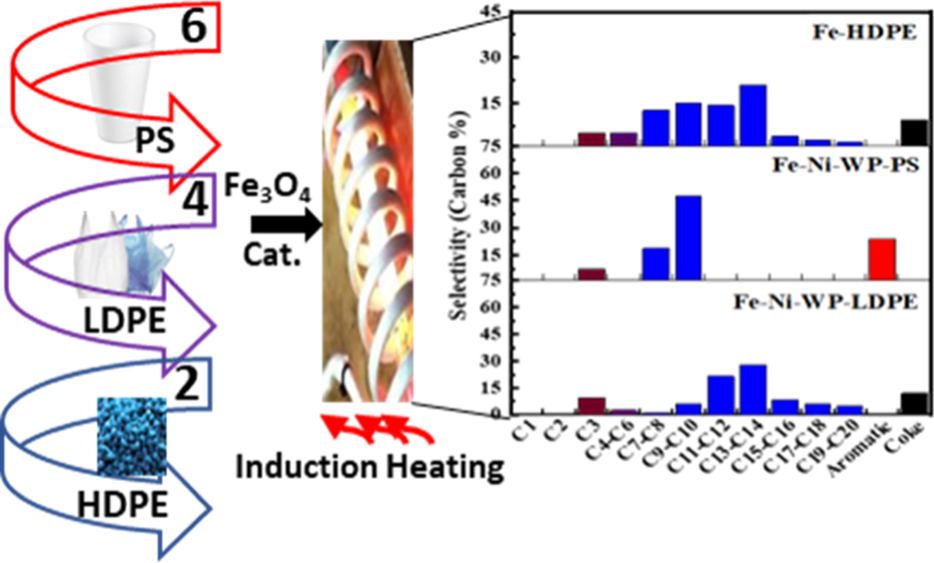

Low- and high-density polyethylene (LDPE/HDPE) have been selectively depolymerized, without added H_2_, to C2–C20 + alkanes/alkenes via energy-efficient radio frequency induction heating, coupled with dual-functional heterogeneous Fe_3_O_4_ and Ni- or Pt-based catalysts. Fe_3_O_4_ was used to locally generate heat when exposed to magnetic fields. Initial results indicate that zeolite-based Ni catalysts are more selective to light olefins, while Ni supported on ceria catalysts are more selective to C7–C14 alkanes/alkenes. LDPE conversions up to 94% were obtained with minimal aromatic, coke, or methane formation which are typically observed with thermal heating. Two depolymerization mechanisms, a reverse Cossee–Arlman mechanism or a random cleavage process, were proposed to account for the different selectivities. The depolymerization process was also tested on commercial LDPE (grocery bags), polystyrene, and virgin HDPE using the Ni on Fe_3_O_4_ catalyst, with the LDPE resulting in similar product conversion (∼48%) and selectivity as for virgin LDPE.

## 1 Introduction

The production of polymers consumes about 5% of the world’s gas and oil, mostly as feedstocks and fuels for polymerization processes, with global production at 400 mmt in 2015, rising at >4%/yr, and 95% of this production was from synthetics.^1^ Despite the substantial amounts of polymers potentially available for re-utilization, it has been estimated that of all synthetic polymers produced since 1950, only 7% have been recycled, compared to 60% which have been discarded (lifetimes > 20 yr), with the rest of these materials either still in use or incinerated.^2^ Polyolefins such as low- and high-density polyethylene (LDPE/HDPE) are among the materials with the lowest rate of decomposition in the environment. Current approaches to recycling plastics have many constraints, making these processes insufficient to curtail the increasing amounts of plastic waste. For example, plastics pyrolysis is limited by economic considerations—it requires high operating temperatures and results in an unwieldy product distribution with little value other than as a low-grade fuel.

There are numerous start-up companies which thermally convert plastics into mixed synthetic light sweet crude.^3^ The yields for these technologies range between 40 and 80%, generally producing higher-molecular-weight products (kerosenes and oils).^4−6^ While little is known about the commercial processes, there have been recent reports discussing the hydrogenolysis of PE over Zr/SiO_2_–Al_2_O_3_ and Ru/CeO_2_.^7,8^ These reactions required high H_2_ pressures (60 bar) to generate a range of C2–C10 hydrocarbons, with products dependent on the temperature, H_2_ pressure, and catalytic metal size/type. To generate lubricant-grade materials, Celik et al.^9^ used Pt-decorated SrTiO_3_ (STO) resulting in an average product of ∼C30 hydrocarbons (280 °C, 11.7 bar H_2_). With Pt/*meso*-SiO_2_, lighter products (C5–C7, C14–C20) can be formed at even lower conversions (250 °C, 13.8 bar).^10^ Conversely, Pt/Al_2_O_3_ and no added H_2_ (280 °C) gave far more alkylaromatics (>50% on a carbon basis) but also >20% heavy waxes.^11^ The demonstrated effect of the STO and *meso*-SiO_2_ supports suggests that other complex metal oxides could direct the depolymerization process based on polymer–substrate interactions.

More acidic supports such as zeolites can also depolymerize polyolefins. While in some cases (Pt-BEA) high H_2_ pressures are required, others have shown that low-pressure reactions can occur over H-ZSM-5 or H-Y zeolites. The process requires higher temperatures (>400 °C),^12^ with generally low selectivities depending on the polymer composition and the zeolite structure. For instance, Miandad et al. found that Faujasite (Si/Al = 9.2) produced mainly char, whereas standard Y-type zeolites generated ∼70% light gases.^13^ Both systems produced primarily aromatics as liquid products, by classical carbenium ion mechanisms initiated by either electron acceptors (Lewis acid)^14^ or proton donors (Brønsted acid).^15^ Similar results were obtained by Kunwar et al. using Y-type zeolites but with lower overall yields (∼40%) compared to those without the catalyst (∼90%).^16^

Microwave or radiofrequency (rf) induction heating has been explored as alternatives to thermal heating since the electromagnetic radiation can directly interact with the polymer and the catalyst.^17,18^ Microwave heating has the advantage that the frequency is tunable to selectively target specific bonds. Unfortunately, microwave-assisted depolymerization processes require the use of solvents to prevent runaway catalyst heating and localized pyrolysis,^19,20^ which results in a carbon product along with the light gases.^21^ To avoid the use of solvents, some groups have turned to induction heating to selectively heat magnetically active materials, typically Fe_3_O_4_, and transfer this energy to neighboring catalysts.^22^ Despite its similarities to microwave heating (heating rate, efficiency, and frequency dependence), there are only a few reports discussing induction heating as an alternative to thermal routes, which can be attributed to multiple factors: magnetically transparent reactors (glass) when necessary,^23,24^ and the fact that the catalyst must interact with the magnetic field and generate significant amounts of heat.^22,24^ While the former requires more expensive reactors, the latter can be overcome with hierarchical catalysts, which include an efficient rf absorber that eliminates the need for conduction/convection to transport heat to the catalyst surface and reduces the generation of hot spots that occur in thermal reactors.^25^ In addition, the rapid and localized heating and the ability to control these temperatures in exo- and endothermic reactions are responsible for the higher catalyst stabilities at elevated temperatures.^25,26^ Finally, it is also believed that the absence of temperature gradients hampers carbonaceous growths typically seen in reactions with high coking rates.^27,28^

To make a lower-temperature and more selective depolymerization process economically preferable to the more entrenched pyrolysis processes, the depolymerization must exhibit high selectivities and yields without being tied to a single type of polymer. Different commercial additives (antioxidants, flame retardants, and plasticizers) and other contaminants present (food residues, green waste, etc.) will require catalysts resistant to coking and poisoning. Herein, we use electromagnetic induction heating (rf) of various Ni-functionalized catalysts to drive the depolymerization of addition polymers (polyolefins) at low bulk liquid temperatures. The goal of this work is the identification of both catalyst and reaction parameters influencing the selectivity for a polyolefin to liquid/gas blend feedstock (alkene/alkane) process.

## 2 Methods

### 2.1 Catalyst Synthesis

Three candidate zeolites already in their H^+^ forms were ion-exchanged first with the K^+^ and then the Ni^2+^ forms using 0.1 M Ni(CH_3_COO)_2_: Beta (BEA), Linde Type-L (LTL), and MFI (ZSM-5, ACS LLC). The exchanged zeolites were dried at 400 °C and calcined in flowing air at 500 °C. The fully exchanged zeolites would contain 2.4 wt % (ZSM-5, Si/Al = 20) or 5.0 wt % Ni (BEA, Si/Al = 8). Additionally, two other silicates (ferrierite, FER, and mesoporous silica, SBA-16) were impregnated with Ni(NO_3_)_2_·6H_2_O due to the lower number of available exchange sites. The silicates were impregnated dropwise with 5 wt % NiO, dried at 100 °C, and calcined at 500 °C in flowing air. An overloaded ZSM-5 (Ni2-ZSM-5) was prepared via dropwise impregnation (to 20 wt % Ni) and calcined similarly. Finally, a Pt(0.5 wt %)–K-ZSM-5 catalyst was made from a K^+^-exchanged ZSM-5 (Si/Al = 29, Zeolyst lot 5534G-1597-94) by leaving it in contact with the zeolite overnight with dilute aqueous platinum diaminodinitrite at pH = 10. The solution was slowly evaporated at 120 °C, followed by a pulse reduction (H_2_ at 400 °C) to give 25% Pt dispersion at RT using H_2_ chemisorption.

A Ni/CeO_2_/ZrO_2_ (Ni–Ce–Zr, 4.7 wt % Ni, 2:1 Ce/Zr atomic ratio) catalyst was synthesized previously^29^ by a molten salt/urea deposition method (80 °C from 0.3 M urea, Ni(NO_3_)_2_·6H_2_O solution, 30:1 solution/solid by weight) and then reduced in 5% H_2_ at 750 °C for 6 h. Nanoparticulate Fe_3_O_4_ (Alfa Aesar, 97%, 50–100 nm, 20–50 m^2^/g) was used as received. A Ni/Fe_3_O_4_ (Fe–Ni, 2.4 wt % Ni) catalyst was made from these nanoparticles by urea deposition of Ni, dried under vacuum at 60 °C, and then reduced in 5% H_2_ at 500 °C for 12 h. 20 wt % Ni on a commercial Ce–Zr–Al support (Ni20-CZA40, from PIDC CZA-40, 1:1 Ce/Zr atomic ratio, 40 wt % Al_2_O_3_) was prepared by two successive incipient wetness impregnations separated by 100 °C dryings, then reduced in 5% H_2_ at 750 °C for 6 h.

A Fe_3_O_4_@CeO_2_ 5:1 (molar) core–shell mixed oxide was synthesized following a modified method of Jiang et al. to produce the Fe_3_O_4_ core.^30^ The CeO_2_ oxide shell was then added by adapting the hydrothermal method of Wei et al.^31^ The particles were washed with ethanol/water after both synthesis steps, instead of drying under N_2_, to avoid oxidation to Fe_2_O_3_. Finally, 5.8 wt % Ni was added by the urea deposition method and dried and reduced by the same way as Fe–Ni to give the catalyst Fe–Ce–CS–Ni.

### 2.2 Thermal Reaction Experiments

Both the H^+^- and Ni^2+^-forms of silicate and zeolite catalysts were used in these experiments. For each run, ∼10–20 mg of catalyst and a typical commercial HDPE (ExxonMobil BA-50 HDPE copolymer, pelletized) were ground together at a 1:1 mass ratio and added to an Al_2_O_3_ sample cup in a TA SDT-600 for thermogravimetry/differential scanning calorimetry (TGA/DSC). From previous work, it was known that the polymer would be both dry and molten by ∼190 °C. The temperature was ramped from 50 °C at 10 °C per min to 190 °C, then from 5 °C to 350 °C, and held for 900 min under a 100 mL/min N_2_ flow.

### 2.3 rf-Activated and Thermally Activated Batch Reaction Experiments

A schematic of the reactor is shown in Figure S1. Briefly, 200 mg of the catalyst/Fe_3_O_4_ powder (1:1 wt ratio) was mixed with 1 g of LDPE polymer (Alfa, 924 kg/m^3^, mp: 105–115 °C). The mixture was loaded in a glass reactor, purged with N_2_, and either exposed to an rf field (300–600 A, 32–64 mT equivalent) or immersed in a heated sand bath (heat supplied by a resistance heater/temperature controller), in both cases for 2 h. A temperature versus magnetic field calibration was performed to correlate the induction heating-induced temperatures. The reaction vessel was cooled for 30 min prior to the collection of gas/liquid products. To calibrate the temperature range in the rf-activated experiments, the Fe_3_O_4_ nanoparticles were mixed with 1-octadecane (bp: 315 °C), *n*-tetracosane (bp 391 °C), or NaCl/ZnCl_2_ salt mixture (mp: ∼ 250–800 °C depending on the salt composition). Alternatively, the Fe_3_O_4_ powders were mixed with hydrothermally grown YVO_4_/Eu^3+^ (3 mol %) nanoparticles (3:1 mixture). Briefly, 1.14 mmol of Y(NO_3_)_3_·6H_2_O and 0.6 mmol of Na_3_C_6_H_5_O_7_·2H_2_O were added dropwise into 0.06 mmol of Eu(NO_3_)_3_·6H_2_O dissolved in 50 mL HNO_3_ solution (12 mM) with continuous stirring for 10 min followed by 1.2 mmol of NH_4_VO_3_ under vigorous stirring. A 1 M NaOH solution was added dropwise until a pH of 9, and the solution was transferred into a 20 mL Teflon-lined autoclave and reacted at 180 °C for 24 h. After cooling naturally, the resultant precipitate was collected and washed with ethanol/water before drying overnight at 100 °C. The photoluminescence intensity was calibrated using a Linkam heating stage connected to an Edinburgh FLS1000 spectrometer. The in situ temperature measurements were collected by placing the Fe_3_O_4_/YVO_4_ mixture in a quartz holder in the center of the rf coil and exposed to the magnetic fields for 2 min prior to the collection of the PL spectra (λ_ex_ = 397 nm; λ_em_ = 575–675 nm).

### 2.4 Product Analysis

The gas atmosphere was sampled during the experiment and analyzed by injection into an SRS RGA200 residual gas analyzer operating in a selective ion mode at the parent *m*/*z* values. Pressure-ion count calibration was based on the injection of standards. The total weight change of the system was used to estimate the conversion to light gases. Other depolymerization products were extracted from the remaining polymer/catalyst mixture with a 90/10 (vol %) 3-methylpentane/DMSO solvent blend for 7 d. The liquid products were then analyzed by gas chromatography–mass spectrometry (GC–MS) on an Agilent 6890 (100 m × 0.25 mm SPB-1 column). The liquid conversion was estimated from the weight change upon drying a sample of catalyst/product mass under vacuum at 170 °C for 7 d. Coke amounts were determined by temperature-programed oxidation (TPO) in air, at 50–250 °C, 10 °C/min, held for 60 min, 10 °C/min to 420 °C, held for 40 min, and 10 °C/min up to 650 °C, held for 60 min. The product selectivity (*S*_*i*_) is defined as
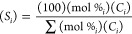
1where *C*_*i*_ is the number of carbons in the compound.

### 2.5 Catalyst Characterization

Surface areas and pore volumes were measured by the BET method (Micromeritics ASAP 2020). TGA/DSC of 1-propylamine (1-PA) was employed to titrate the Brønsted sites, as discussed by Gorte^32,33^ and Price and Dooley,^34^ based on desorption temperature shifts and decreases in adsorbed amounts associated with replacement of H^+^ by Ni^2+^.

## 3 Results and Discussion

### 3.1 Thermal Reactions

**Figure 1:**
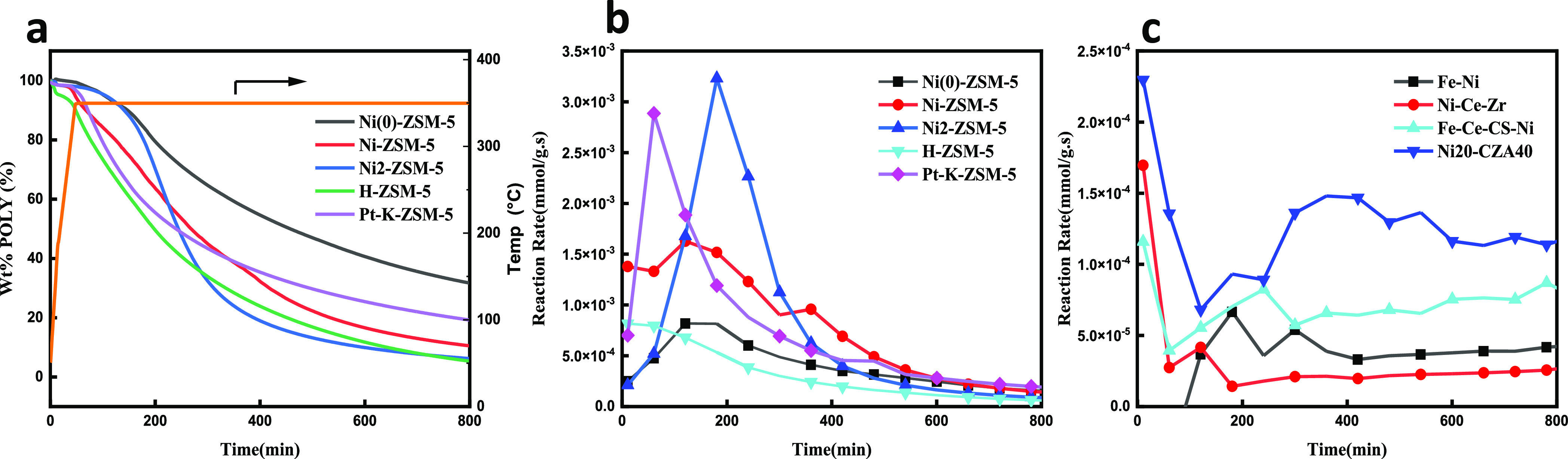
Weight loss and rate variation curves: (a) HDPE wt loss curves over modified ZSM-5 catalysts heated to 350 °C as a function of time, (b) temporal rate variation in TGA/DSC analyses for the zeolites catalysts, and (c) temporal rate variation in TGA/DSC analyses for the metal oxide catalysts.

Initially, the catalysts were thermally screened (TGA/DSC) using HDPE/catalyst blends. Catalysts were characterized based on their overall reaction rates (mass change, eq S1) and heat flux (indicative of selectivities to lower MW products, eq S2). The results of these screening experiments are shown in Table 1. A blank run (no catalyst) showed no polymer weight loss at >150 °C, with minor losses at lower temperatures due to drying. The heat flux is calculated for all times after the polymer melting is complete and the DSC baseline is smooth (>200 °C). As almost all the weight loss occurred during the 350 °C hold (Figure 1a), the rates can be considered typical of that temperature.

**Table 1:** Depolymerization Rate and Selectivity Data (TGA/DSC) and Morphological Characterization for Various Zeolite/Metal Oxide Catalysts

catalyst	10^4^ × rate (mmol g_cat_^–1^ s^–1^)	heat/wt poly (J/g)	surface area (m^2^/g)	pore volume (cm^3^/g)
Ni-BEA	0.79	–228	480	0.28
H-BEA	3.3	–1720		
Ni–ZSM-5	7.2	4840	310	0.36
Ni(0)–ZSM-5	3.7	5960		
Ni2–ZSM-5	7.1	410	300	0.22
H-ZSM-5	2.9	8190	320	0.32
Ni-FER	0.11	–254	49	0.17
H-FER	3.0	724		
Ni-LTL	1.7	–5260	550	0.31
H-LTL	4.1	820		
Ni-SBA	3.1	5360	480	0.37
Fe_3_O_4_	0.81	7720	33	0.11
Ni–Ce–Zr	0.32	–3250	26	0.12
Fe–Ce–CS–Ni	0.71	–1790	37	0.16
Fe–Ni	0.24	318	4.9	0.025
Ni20–CZA40	1.3	1840	79	0.49
Pt–K-ZSM-5	7.1	–2580	370	0.25
Pt complex	63	9820	N/A	

This method assumes that all low MW products (<C20) will be vaporized in the N_2_ flow. Therefore, it is hypothesized that the measured weight loss is proportional to the rate of depolymerization to useable products. Additionally, the heat per weight of polymer is a measure of the overall, average heat of the reactions. While it is not possible to distinguish the formation of light gases, aromatics, or coke from other products based on the average heat flux, this metric can distinguish endothermic from exothermic reactions. The highly endothermic reactions are expected to correspond to a mixture rich in light alkenes such as ethylene (Δ*H*_depoly_ = 3825–3875 J/g).^35^ Less endothermic values correspond to a mixture richer in mid-range alkenes (the heat of reaction for C_20_H_40_ to two moles of decene is 640 J/g).^36^ However, exothermic values suggest the formation of aromatics/coke and the concomitant hydrogenation to alkanes. Additionally, there are enthalpy changes associated with the catalyst itself (phase transformations, surface reconstructions, oxidation, etc.) that affect the measured heat flux.

An initial screening of the reaction rates shows that the Ni-modified ZSM-5 catalysts demonstrate much higher activities than the other zeolites. It appears that a coordinated Ni (Ni-ZSM-5) structure plays an important role in the decomposition process. Reducing this catalyst (in 5% H_2_ at 350 °C, Ni(0)-ZSM-5 in Table 1), significantly decreased the activity (by ∼50%). The higher heat flux of the reduced sample is likely due to some oxidation of the Ni species during the TGA/DSC experiment. Deposition of extra Ni onto the catalysts (Ni2-ZSM-5) has negligible impact on the overall reaction rate while significantly decreasing the heat flux, suggesting the formation of more alkanes or aromatics. On the other hand, the Pt-exchanged zeolite (Pt–K-ZSM-5) exhibits high, exothermic reaction rates. In addition to coking or aromatic formation, Pt–zeolite catalysts are well known for their hydrocracking capability (exothermic). The other zeolites gave lower reaction rates (<3 × 10^–4^ mmol g^–1^ s^–1^) with exothermic or slightly endothermic heat fluxes (<1000 J/g) for the H^+^- and Ni^2+^-modified forms, except for Ni–SBA. Conversely, the reaction rates (and surface area) for the metal oxide catalysts were low. However, the endothermic heat flux for the Fe_3_O_4_ catalyst was greater than all but the Pt organometallic complex and H-ZSM-5. The high endothermic flux indicates the formation of some heavy non-volatile hydrocarbons.

To understand the depolymerization process over the ZSM-5 and metal oxide catalysts throughout the experiment, time-dependent reaction rates (Figure 1b,c) were extracted. The polymer conversion at any time is approximately 100 wt % polymer (Figure 1a). The rates for the ZSM-5 catalysts (Figure 1b,c) build to a maximum as the temperature approaches 350 °C and then decrease with time. Alternatively, an initial decrease (Ce-based oxides) or increase (Fe–Ni) in reaction rates for the oxides is attributed to the removal of surface hydroxyls or substrate oxidation, respectively. The decrease in rate over time is partly due to the consumption of polymer but also possibly due to coke formation and pore blockage. Without larger-scale experiments and spent catalyst characterizations, these two possibilities cannot be distinguished. However, the heat fluxes are relatively stable for all catalysts, suggesting a continuous depolymerization process. From these experiments, it is seen that the exchanged zeolites are more active after an initial induction period, a period which can be attributed to slow polymer pore diffusion. These diffusional resistances are less for the large-pore metal oxides; however, the decreased reaction rates for the metal oxides compared to the zeolites are in keeping with the relative surface areas (10-fold decrease for the Ce-based oxides compared to the zeolites, Table 1). To obtain a Ni–CeO_2_-based catalyst with somewhat higher surface area and pore volume, a commercial support containing 40 wt % Al_2_O_3_ (Fe–Ni20–CZA40) was used, showing higher reaction rates (2–5 times) than the in-house catalysts.

As a comparison, the activity of a homogeneous Pt catalyst [Pt(divinyltetramethylsiloxane), 2.25 wt % in xylene] was measured. One would expect the soluble homogeneous Pt catalyst to give even higher rates due to more intimate contact between the polymer and the catalyst and the overall cracking activity of Pt compared to Ni. Xylene does not impact the reaction rate or heat flux calculations since the solvent evaporates (bp: 139 °C) before the polymer melting point is reached. The average reaction rate is much higher than the heterogeneous catalysts (Table 1). At longer times, the rates for the Pt complex are comparable to those of Ni–ZSM-5-based catalysts. Regardless of the catalyst, the observed reaction rates would require long reaction times or large quantities of catalyst (50,000 kg of Ni–ZSM-5 per kg per s polymer reacted) to be commercially viable. As such, alternative approaches must be explored to enhance the reaction rates.

### 3.2 rf-Activated Reactions

**Figure 2:**
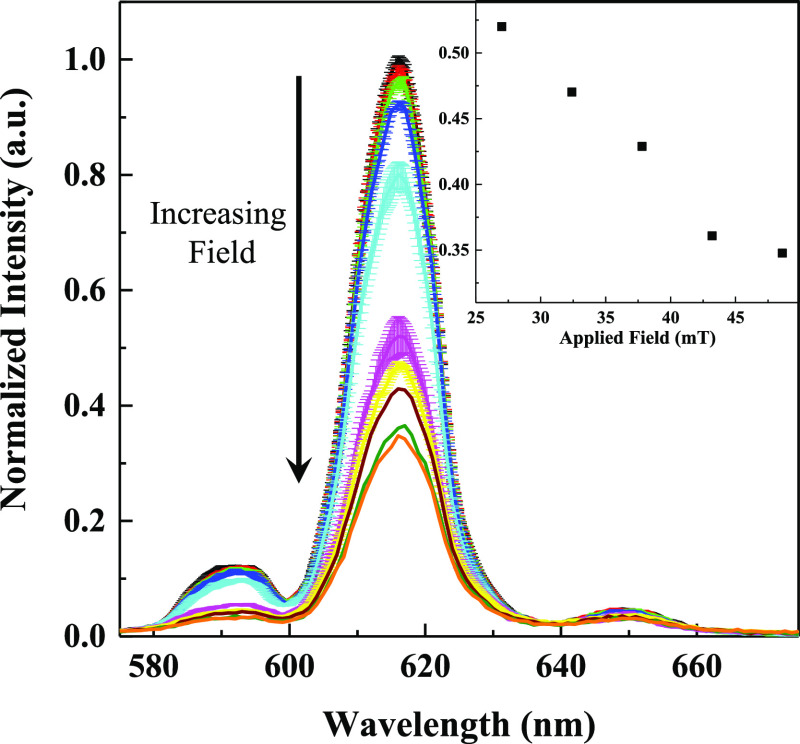
PL response of the Fe_3_O_4_/YVO_4_/Eu^3+^ mixture under applied rf fields. The inset highlights the linear response of the normalized intensity at high applied fields (200–400 °C).

Induction heating was employed as an alternative to thermal heating due to the increased heat-transfer efficiencies and the ability to locate the heat at the active catalyst site. Before the depolymerization reactions could proceed, it was necessary to calibrate the reaction temperature. To calibrate these field-dependent temperatures, the Fe_3_O_4_ powder was mixed with various heavy hydrocarbons or salt mixtures and exposed to magnetic fields up to 64 mT. The mixtures were visually observed for solvent boiling [1-octadecane (315 °C@38 mT)/*n*-tetracosane (391 °C@59 mT)] or salt melting [ZnCl/NaCl (420 °C@64 mT)]. As a secondary confirmation, a Fe_3_O_4_/YVO_4_/Eu^3+^ mixture (3:1 by wt) was used to estimate the temperature based on the photoluminescence intensity. The Eu^3+^ intensity is known to be inversely proportional to the temperature.^37^ The PL measurements increased linearly above 25 mT (Figure 2 and Tables S1 and S2) and reached an estimated surface temperature of ∼420 °C at 64 mT, comparable to those required for polymer pyrolysis/degradation.^12,13,15,16,38,39^

**Figure 3:**
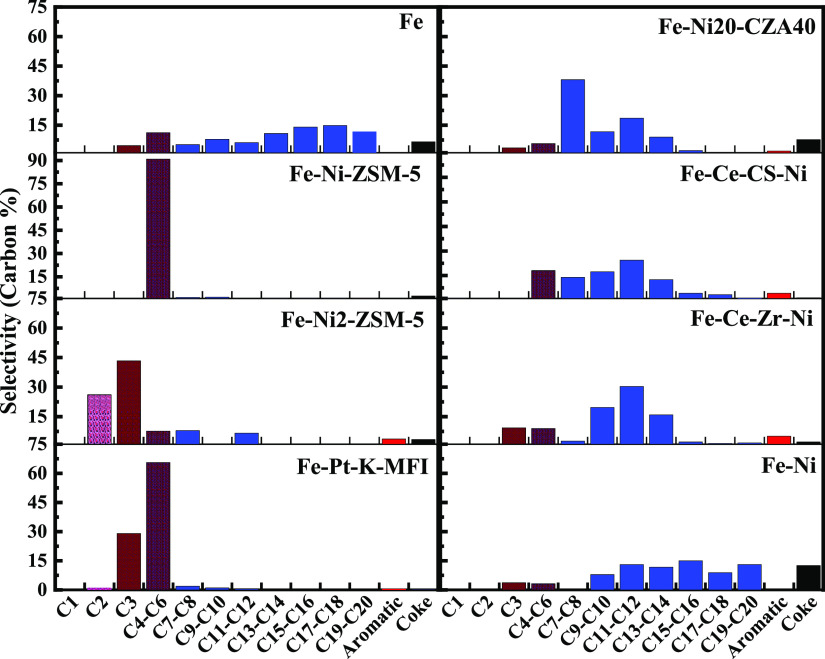
rf (64 mT field)-initiated LDPE depolymerization for various zeolite (left) and non-zeolite (right) catalysts. The different colors are just an aid to the eye. The “Fe” in all but Fe–Ni denotes that 50 wt % of the catalyst is Fe_3_O_4_ nanoparticles. For Fe–Ni, there are 97.6 wt % Fe_3_O_4_ nanoparticles; 115 mg of total catalyst.

Two types of catalysts were chosen for induction heating based on the TGA screening results, modified ZSM-5 (Ni–ZSM-5, Ni2–ZSM-5, Pt–K-MFI) and CeO_2_-based catalysts. Commercial Fe_3_O_4_ powder was added to the reactor to act as a magnetic susceptor. Conversions to liquid and gas products are reported in Tables 2 and S3, and the product distributions are reported on a carbon % basis in Figure 3. Some H_2_ was also observed (Table S3, as a percentage of the conversion to gas). The gas product RGA and liquid GC–MS scans are shown in Figures S2 and S3. The RGA scans suggest that CH_4_ formation is minimal (Figure S2). Similar results for conventional thermally driven reactions using the Ni2-ZSM-5 catalyst are also given in Table S3 with the selectivities reported in Figure S4. On comparing the rf and thermal results at a similar surface temperature (420 °C), the observed first-order rate constant was found to be 25 times faster for the rf-activated reaction. If the comparison was made on a bulk (fluid) temperature basis, the comparison would be even more in favor of rf activation. Relatively less H_2_ was also produced under rf conditions (Table S3).

The zeolite-based catalysts produced significantly more light gases and light liquids, with the metal oxides generating more diesel-range products. The Ni2–ZSM-5 catalyst generated mostly C2–C3 light gases compared to Ni–ZSM-5 and Pt–K-MFI, which produced a lot of C4–C5. For Pt–K-MFI, these light gases/liquids are primarily olefins based on preliminary GC–MS analysis (Figure S3). The Ce-based catalysts tended to generate lower-molecular-weight liquids than Fe or Fe–Ni. As a comparison, the Fe and Fe–Ni samples were run at higher Fe/polymer ratios (1:5 Fe/LDPE) which mimic the catalyst/LDPE ratios used in the other experiments (Figure S5) but would give higher temperatures since there is more Fe_3_O_4_. The product distributions in this case shift to higher concentrations of light gases, suggesting that the cleavage process generates lower-molecular-weight hydrocarbons at higher temperatures. While there was no effort to exactly quantify the relative amounts of alkenes/alkanes, the liquid products are roughly in the 1:1–2:1 range. Similar to the TGA/DSC results, Ni on the commercial Al_2_O_3_–CeO_2_–ZrO_2_ support gave a higher total conversion (by 10%).

### 3.3 Catalyst Characterization

**Figure 4:**
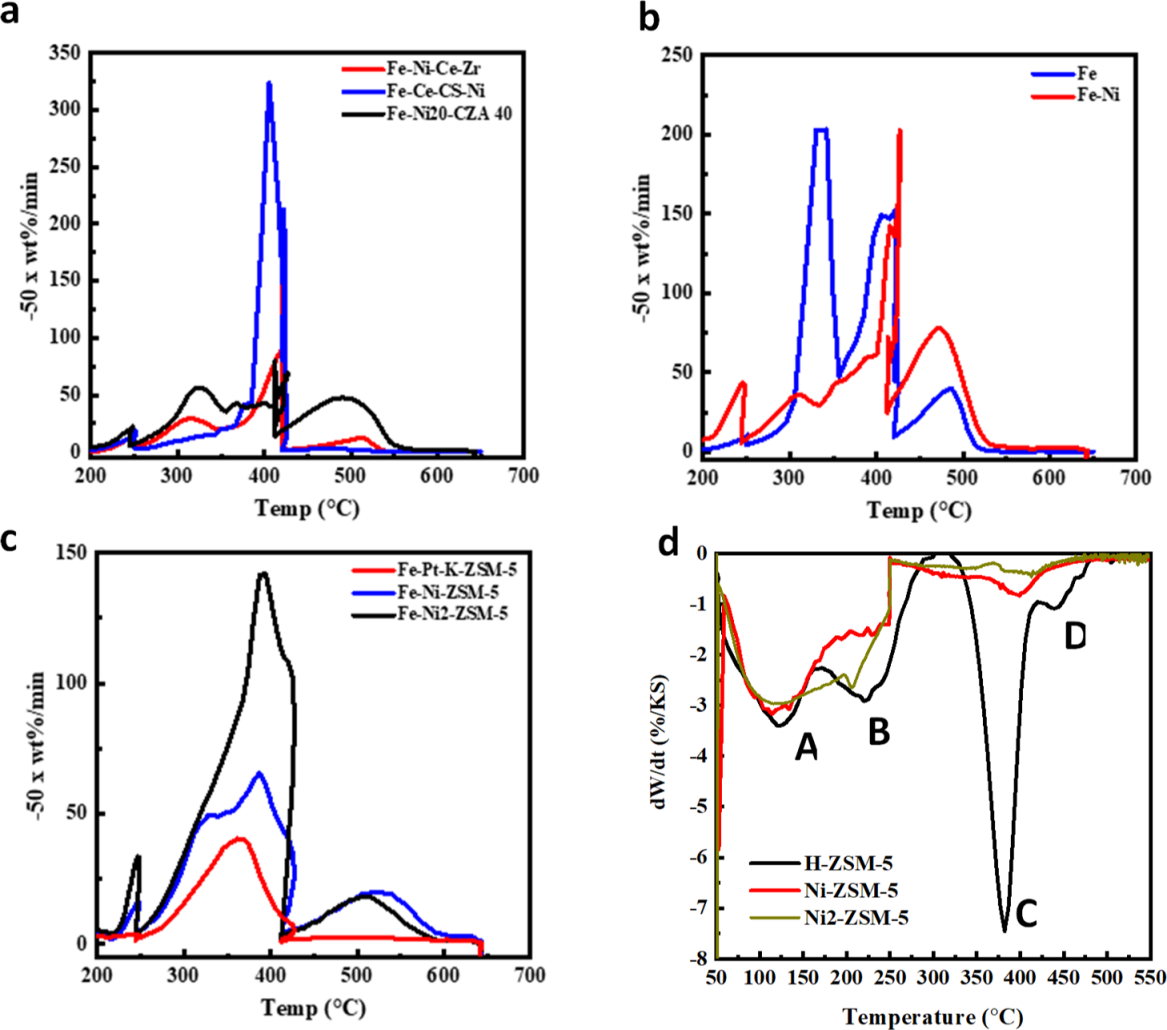
Coke and acid site analysis. TPO weight derivatives for used, extracted catalysts after LDPE depolymerization for (a) catalysts containing CeO_2_, (b) Fe_3_O_4_ and Ni-supported on Fe_3_O_4_, and (c) zeolite catalysts. The presence of coke is seen from the peaks at above 420 °C. (d) Differential thermal analysis of amine desorption of the three ZSM-5-based catalysts. Peaks A and B arise from the desorption of weakly adsorbed 1-PA not on Brønsted sites, peak C from the Hofmann elimination of 1-PA to propene and NH_3_ on Brønsted sites, and peak D from dehydrogenation chemistry on strong Lewis sites, normally associated with extra-framework Al^3+^. The Si/Al molar ratio obtained by magic angle spinning-NMR spectroscopy for H-ZSM-5 is 20, while the ratio computed from these data is 21. The small “C” peak for Ni–ZSM-5 corresponds to <10% residual H^+^.

The used, extracted catalysts were analyzed by TPO to estimate how much of the polymer was converted to heavier aromatic or graphitic (“coke”) material (Figure 4a–c). The coke conversions were calculated using eq S3 and reported in Table 2. There was a small peak at <200 °C (not shown) due to solvent vaporization. The peaks between 220 and 420 °C are attributed to the oxidation of the residual polymer with the higher temperature peaks (>420 °C) arising from coke/heavy aromatic oxidation. This was checked by running both LDPE and HDPE standards where the unreacted polymer and the catalyst were ground together. Additionally, the Fe_3_O_4_ is oxidized to Fe_2_O_3_ during the oxidation process between 400 and 600 °C. However, the contribution to the weight changes caused by this oxidation is negligible, calculated as only 0.1% maximum. As a secondary confirmation of the presence of some heavy carbon products, Raman spectroscopy was performed on a select set of used samples to identify the presence of a small graphitic G0 band (1595–1605 cm^–1^).

**Table 2:** LDPE Depolymerization Using a 64 mT Induction Field for 2 h under 1 atm N_2_^a^

catalyst	liquid conversion (wt %)	gas conversion (wt %)	coke conversion (wt %)	aromatics^b^ (carbon %)
Fe–Ni-ZSM-5	2	75	2.1	0.81
Fe–Ni2-ZSM-5	4	54	2.0	3.7
Fe–Pt–K-MFI	2	80	0.33	0.43
Fe–Ce–CS–Ni	24	16	0.56	4.5
Fe–Ni–Ce–Zr	26	26	1.1	5.1
Fe–Ni20–CZA40	43	19	5.2	1.9
Fe–Ni	35	15	7.1	0.0
Fe	26	19	3.2	1.2

a Liquid, gas, and coke conversions are on weight basis with aromatic (one and two rings) selectivity reported on a carbon % basis. Heavier than two-ring aromatics have been identified with “coke”.

b Single and two-ring aromatics.

Finally, to understand the nature of the surface sites within the ZSM-5 catalysts, the Brønsted/Lewis acid site concentrations and strengths were quantified. The split between Brønsted, weak Lewis, and strong Lewis acid sites in the zeolites was assessed using a 1-propanamine (1-PA) desorption method pioneered by Gorte^32,33^ and modified for metal-exchanged materials by Price and Dooley.^34^ 1-PA accurately titrates Brønsted sites in H-form zeolites and can provide reasonable estimates of residual Brønsted sites in metal-exchanged zeolites because desorption peaks associated with 1-PA on the ionic metals shift to higher or lower temperatures. This titration also detects framework atoms that might give rise to weaker acid sites and their departure from the framework.^40^ An example analysis for the three ZSM-5-based catalysts is shown in Figure 4d. The low-temperature peaks (peaks A and B) are associated with weak Lewis acid interactions with 1-PA. The 1-PA associated with H^+^ in the zeolite framework desorbs at 350–410 °C (peak C). Replacing these with Ni^2+^ results in a sharp decrease of this peak, essentially disappearing for the overloaded Ni2–ZSM-5. Unlike the case for certain exchanged metals (Ga^+^ or Al^3+^, e.g., refs (34) and (41)), there is no evidence for the generation of strong Lewis sites by Ni^2+^ (peak D in Figure 4d). The total amounts of 1-PA adsorbed decrease even at the lower temperatures (peak B), suggesting weaker Lewis acidity associated with these metal-exchanged (or in the case of Ni2–ZSM-5, exchanged Ni but also additional NiO). However, the coordination of the active Ni is not the same as in NiO because the Ni–SBA catalyst, with Ni-impregnated high surface area SBA-16, showed no activity. This suggests that some degree of Ni–zeolite coordination at framework sites is necessary for a functioning depolymerization catalyst of this type, as also seen with the poorer activity of the reduced Ni(0)–ZSM-5.

### 3.4 Discussion

Mostly Ni-based catalysts were chosen for depolymerization under the hypothesis that catalysts which can oligomerize low-molecular-weight olefins should also catalyze the reverse reaction. The only problem with the catalysts containing Ni-impregnated CeO_2_ is their lower activity. The CeO_2_-based catalyst with a high wt % Ni (Ni20–CZA40) gave more coke than the zeolite-based catalysts, but, as expected, the CeO_2_-based catalysts with only a few wt % Ni gave very little coke.^29,42^ The cleavage mechanism of the Ce-based metal oxides produced diesel-range hydrocarbons of a fairly narrow molecular-weight distribution with minimal light gases, giving these catalysts an advantage over the zeolites if diesel is desired. However, the two experiments with Ni/Fe_3_O_4_ (Fe–Ni) showed that it is also possible to control the product distribution based on applied heat (higher surface temperature), even with a simpler catalyst.

It is hypothesized that the differences in product distributions for the zeolites compared to the metal oxides have resulted from cleavage nearer to terminal carbon groups within the zeolite pores. This is not an artifact of higher conversion. Note that the product distribution for Ni20–CZA40 is still skewed toward heavier liquid products, while its activity is comparable to that of Ni–zeolite catalysts. López et al.^43^ postulated that for zeolite-based catalysts the depolymerization reaction generally occurs on the zeolite crystal surfaces rather than within the pores, due to diffusion limitations. However, this is somewhat contrary to previous literature regarding pore diffusion of long-chain molecules in zeolites^44^ and in mesoporous SiO_2_.^10^ Can polyethylene chains enter the zeolite pores? We determined a cutoff minimum effective diffusivity (*D*_e_) of 3 × 10^–15^ m^2^/s for spherical particles (*d*_p_ = 2 μm) of the type used here
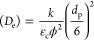
2assuming a Thiele modulus (φ) of 1, a rate constant *k* of 2.4 × 10^–3^ s^–1^ (calculated as shown in eq S4 of the Supporting Information), and a catalyst/polymer ratio (ε_c_) of 0.1. The bulk diffusivity for polyethylene (in the melt, over a wide range of molecular weights, branching levels, and grades) at 200 °C is between 2 × 10^–14^ and 3 × 10^–12^ m^2^/s.^45−48^ In its random coil state, no polymer molecule could penetrate a microporous material such as a zeolite. The radius of gyration for PE (similar to its hydrodynamic radius *R*_H_) is still >4 nm at 150 °C,^49^ and ratios of *R*_H,polymer_/*R*_pore_ > ∼0.2–0.4 are known to reduce *D*_e_’s to effectively zero.^50,51^ However, the strong heats of adsorption in the zeolites (they increase linearly with carbon number for most zeolites and silicas), and the gains in conformational entropy upon “flattening” the chains to a more planar zig-zag configuration, drive the diffusive process at high temperatures in microporous materials, with specific repulsive interactions absent. For zeolites, the intraparticle diffusivities of the alkane/alkene families approach a constant minimum (>10^–11^ m^2^/s) with respect to molecular weight even at short chain lengths, at temperatures much lower than those used here.^52,53^ Recent solid-state NMR measurements for HDPE in *meso*-SiO_2_ (1.5 nm pores) suggest even higher diffusivities, ∼2 × 10^–9^ m^2^/s, at 114 °C.^10^ This type of conformational change for alkyl chains is well known in catalysis; for example, for triglyceride hydrogenation, measured *D*_e_’s can actually be 2–6 times greater than bulk diffusivities (due to surface diffusion of planar zig-zag conformers)^54^ and in size-exclusion chromatography polyolefins routinely penetrate pores far smaller than their presumed hydrodynamic radii. We conclude that for the rates observed here, the reactions are not diffusion-limited and that the polymer chains can penetrate the pores of ZSM-5 to some extent.

We expect differences in reactivity for purely ion-exchanged versus extra-framework Ni even using the same zeolite (ZSM-5), as observed above (Table 2, Figure 3). Specifically, the Ni^2+^ (or slightly less electropositive) coordination within the zeolite dictates electron back donation to the antibonding states,^55,56^ affecting the available *d*-band states for polymer interaction.^57^ The Ni^2+^-exchanged zeolites (at least in the AFI and LTA topologies) are known to drive polymerization by converting to immobilized alkyl complexes apparently capable of both β-hydride elimination and olefin insertion in a likely Cossee–Arlman-type mechanism.^58,59^ DFT calculations have shown that such immobilized Ni^2+^ mimics homogeneous catalysts,^59^ in some cases achieving a preferred (for polymerization) square-planar coordination.^58^ The zeolite structure also promotes chain growth via diffusion-limited processes.^60,61^ Therefore, highly dispersed (via Si–O–Al exchange sites) and immobilized Ni^2+^ (and Pt^+^) sites within the zeolite should be able to reversibly depolymerize by a reverse Cossee–Arlman mechanism. All of these M-exchanged zeolites give high selectivities to lighter carbon products, as might be expected from such a mechanism. However, Ni-ZSM-5 shows residual strong acid sites (Brønsted acid)^60,62^ by 1-PA titration, which could account for the lower ethylene selectivity.

In contrast, the Ni^2+^-doped rare earth oxides and Ni/Fe_3_O_4_ must catalyze depolymerization by an entirely different mechanism. It has been found that for other supported organometallic complexes such as Zr oxyhydrides/SiO_2_ scission is almost random in nature at 150 °C.^63^ Some product selectivity occurs with these samples because there were essentially no products observed above C20 for the Ni–CeO_2_-based catalysts. Extended extraction times and extractions with a slightly better solvent for HDPE (*o*-xylene) also gave no higher-molecular-weight products. On the other hand, the Fe and Fe–Ni did generate higher-molecular-weight products, suggesting a more random cleavage process. Therefore, the Ni–CeO_2_ product distributions, centered around C7–C14, reflect intrinsic depolymerization activity of these catalysts, instead of purely random scission. Whether this arises from a diffusional cutoff related to pore size and/or certain preferred conformations of >C20 species in larger pores is an open question.

We can compare our process to that of a typical microwave-initiated depolymerization for HDPE.^21^ In this process, the 1:1 FeAlO_*x*_/HDPE catalyst mixture generated temperatures starting at 350 extending to >400 °C during a run. For the first cycle, they obtained gas yields of ∼65% (mass basis), with most of the remaining product detected as coke or iron carbide. The gas was composed of 80 vol % H_2_ and 5–10% CO with the remainder consisting of CH_4_, CO_2_, and C2+ gases. The different mechanisms seen between the microwave process and our rf-activated depolymerization can be attributed to the differences in how microwave versus rf radiation interacts with the polymer and the catalyst. In the rf-driven process, there is localized hysteresis heating of the Fe_3_O_4_, followed by the activation of C–C bonds within the hydrocarbon backbone instead of direct activation of the hydrocarbons.

**Figure 5:**
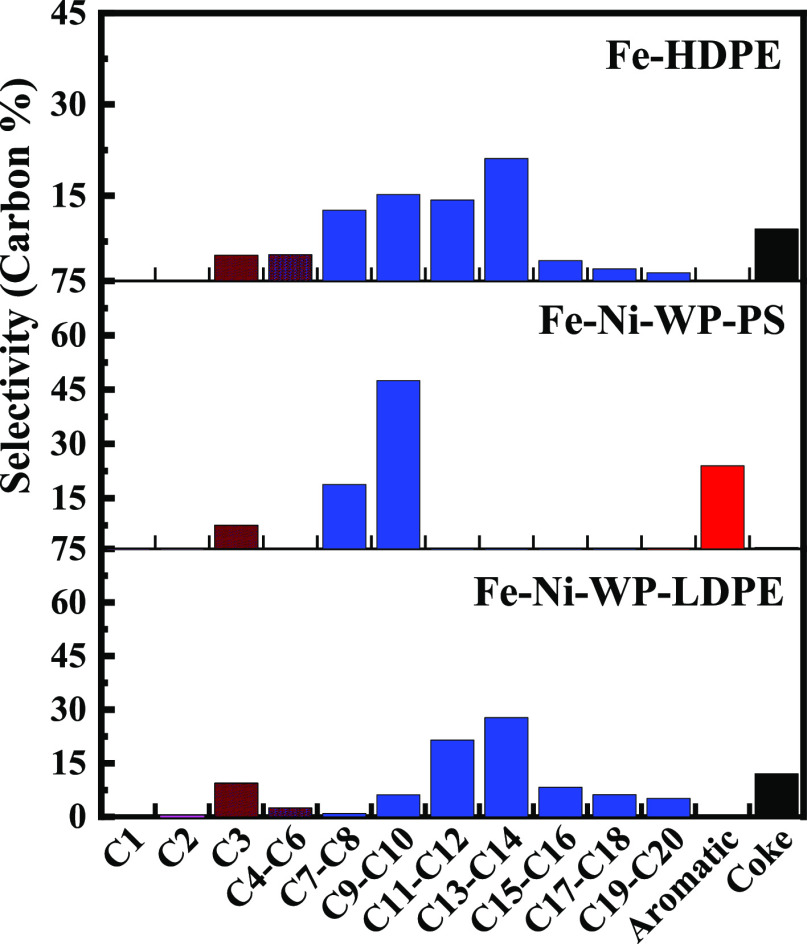
rf-initiated commercial LDPE reaction. Product distribution of commercial LDPE (WP-LDPE) and polystyrene (WP-PS) over Fe–Ni catalysts and virgin HDPE over the Fe catalyst exposed to 64 mT rf field for 2 h.

Finally, depolymerizations of commercial LDPE (grocery bags), commercial polystyrene (Styrofoam), and virgin HDPE were performed over the Fe–Ni catalyst as proof-of-concept experiments. For commercial LDPE, the depolymerization conversion after 2 h for a 115:1000 catalyst/polymer wt ratio was 54% (28.4% liquid, 19.4% gas, and 6.5% coke) with product selectivities shown in Figure 5. This conversion and the selectivities are similar to the virgin polymer. The conversion for the commercial polystyrene was 33% and that of the virgin HDPE was 48%. The HDPE depolymerization has a similar selectivity as the LDPE, with the products centered around C13–C14 but generated more light liquid products. The process appears to work for all common polyolefins.

## 4 Conclusions

In summary, LDPE and HDPE depolymerization was studied using thermal and induction heating of Ni-activated zeolites and metal oxides, without added H_2_. The thermal decomposition process agreed well with previous results showing the onset of polymer decomposition around 350 °C, regardless of the catalyst structure but requiring significantly long reaction times for high conversion. Alternatively, the rf-driven process resulted in high conversions (up to 94%) after exposure to 64 mT fields for 2 h. The surface temperatures were calibrated using the mp/bp of different solvents. The depolymerization process was shown to be dependent upon the catalyst structure, with no observable diffusional limitations, proceeding either through a reverse Cossee–Arlman (zeolites), selective cleavage (CeO_*x*_), or a random cleavage (Fe) route. As such, the resulting product distributions ranged from mainly light gases (C2–C5), diesel-range products (C7–C14), or a wider range of liquids (C8+). Finally, the depolymerization of commercial LDPE (grocery bags) over a Fe–Ni catalyst produced mainly C10–C20 alkanes/alkenes. The novelty of this work is that the rf-driven depolymerization process allows for controlled (minimal CH_4_ and H_2_) and product-tunable decomposition of virgin and commercial grade polyolefins to rapidly (at least 25 times faster than the corresponding thermally driven reaction) produce either light gases or diesel-grade products with no added H_2_. Little coke is produced, even at high conversions. The process has the potential to upcycle a range of commercial plastics into monomers or specialty chemical feedstocks without employing either noble metals or H_2_ feeds as an economically viable alternative to the current recycling methods.

## Supporting Information Available

Calibration of rf temperature, schematic of the reactor system, reaction rate equations, and product characterization (PDF)

## References

[ref1] Geyer R.; Jambeck J. R.; Law K. L. Production, use, and fate of all plastics ever made. Sci. Adv. 2017, 3, e1700782 10.1126/sciadv.1700782. 28776036 PMC5517107

[ref2] Forum W. E. The World’s Plastic Problem in Numbers. https://www.weforum.org/agenda/2018/08/the-world-of-plastics-in-numbers (accessed July 12, 2019).

[ref3] Closed Loop Partners. Advancing Circular Systems for Plastics. https://www.closedlooppartners.com/research/advancing-circular-systems-for-plastics/ (accessd July 2020).

[ref4] Shah J.; Jan M. R.; Mabood F.; Jabeen F. Catalytic pyrolysis of LDPE leads to valuable resource recovery and reduction of waste problems. Energy Convers. Manage. 2010, 51, 2791–2801. 10.1016/j.enconman.2010.06.016.

[ref5] Scott D. S.; Czernik S. R.; Piskorz J.; Radlein D. S. A. G. Fast pyrolysis of plastic wastes. Energy Fuels 1990, 4, 407–411. 10.1021/ef00022a013.

[ref6] Liu Y.; Qian J.; Wang J. Pyrolysis of polystyrene waste in a fluidized-bed reactor to obtain styrene monomer and gasoline fraction. Fuel Process. Technol. 2000, 63, 45–55. 10.1016/s0378-3820(99)00066-1.

[ref7] Oya S.-i.; Kanno D.; Watanabe H.; Tamura M.; Nakagawa Y.; Tomishige K. Catalytic production of branched small alkanes from biohydrocarbons. ChemSusChem 2015, 8, 2472–2475. 10.1002/cssc.201500375. 26097221

[ref8] Nakaji Y.; Nakagawa Y.; Tamura M.; Tomishige K. Regioselective hydrogenolysis of alga-derived squalane over silica-supported ruthenium-vanadium catalyst. Fuel Process. Technol. 2018, 176, 249–257. 10.1016/j.fuproc.2018.03.038.

[ref9] Celik G.; Kennedy R. M.; Hackler R. A.; Ferrandon M.; Tennakoon A.; Patnaik S.; LaPointe A. M.; Ammal S. C.; Heyden A.; Perras F. A.; Pruski M.; Scott S. L.; Poeppelmeier K. R.; Sadow A. D.; Delferro M. Upcycling Single-Use Polyethylene into High-Quality Liquid Products. ACS Cent. Sci. 2019, 5, 1795–1803. 10.1021/acscentsci.9b00722. 31807681 PMC6891864

[ref10] Tennakoon A.; Wu X.; Paterson A. L.; Patnaik S.; Pei Y.; LaPointe A. M.; Ammal S. C.; Hackler R. A.; Heyden A.; Slowing I. I.; Coates G. W.; Delferro M.; Peters B.; Huang W.; Sadow A. D.; Perras F. A. Catalytic upcycling of high-density polyethylene via a processive mechanism. Nat. Catal. 2020, 3, 893–901. 10.1038/s41929-020-00519-4.

[ref11] Zhang F.; Zeng M.; Yappert R. D.; Sun J.; Lee Y.-H.; LaPointe A. M.; Peters B.; Abu-Omar M. M.; Scott S. L. Polyethylene upcycling to long-chain alkylaromatics by tandem hydrogenolysis/aromatization. Science 2020, 370, 437–441. 10.1126/science.abc5441. 33093105

[ref12] Chandrasekaran S. R.; Kunwar B.; Moser B. R.; Rajagopalan N.; Sharma B. K. Catalytic Thermal Cracking of Postconsumer Waste Plastics to Fuels. 1. Kinetics and Optimization. Energy Fuels 2015, 29, 6068–6077. 10.1021/acs.energyfuels.5b01083.

[ref13] Miandad R.; Barakat M. A.; Rehan M.; Aburiazaiza A. S.; Ismail I. M. I.; Nizami A. S. Plastic waste to liquid oil through catalytic pyrolysis using natural and synthetic zeolite catalysts. Waste Manage. 2017, 69, 66–78. 10.1016/j.wasman.2017.08.032. 28882427

[ref14] Rizzarelli P.; Rapisarda M.; Perna S.; Mirabella E. F.; La Carta S.; Puglisi C.; Valenti G. Determination of polyethylene in biodegradable polymer blends and in compostable carrier bags by Py-GC/MS and TGA. J. Anal. Appl. Pyrolysis 2016, 117, 72–81. 10.1016/j.jaap.2015.12.014.

[ref15] De Stefanis A.; Cafarelli P.; Gallese F.; Borsella E.; Nana A.; Perez G. Catalytic pyrolysis of polyethylene: A comparison between pillared and restructured clays. J. Anal. Appl. Pyrolysis 2013, 104, 479–484. 10.1016/j.jaap.2013.05.023.

[ref16] Kunwar B.; Chandrasekaran S. R.; Moser B. R.; Deluhery J.; Kim P.; Rajagopalan N.; Sharma B. K. Catalytic Thermal Cracking of Postconsumer Waste Plastics to Fuels. 2. Pilot-Scale Thermochemical Conversion. Energy Fuels 2017, 31, 2705–2715. 10.1021/acs.energyfuels.6b02996.

[ref17] Achilias D. S.; Redhwi H. H.; Siddiqui M. N.; Nikolaidis A. K.; Bikiaris D. N.; Karayannidis G. P. Glycolytic depolymerization of PET waste in a microwave reactor. J. Appl. Polym. Sci. 2010, 118, 3066–3073. 10.1002/app.32737.

[ref18] Siddiqui M. N.; Achilias D. S.; Redhwi H. H.; Bikiaris D. N.; Katsogiannis K.-A. G.; Karayannidis G. P. Hydrolytic depolymerization of PET in a microwave reactor. Macromol. Mater. Eng. 2010, 295, 575–584. 10.1002/mame.201000050.

[ref19] Milovanović J.; Rajić N.; Romero A. A.; Li H.; Shih K.; Tschentscher R.; Luque R. Insights into the Microwave-Assisted Mild Deconstruction of Lignin Feedstocks Using NiO-Containing ZSM-5 Zeolites. ACS Sustainable Chem. Eng. 2016, 4, 4305–4313. 10.1021/acssuschemeng.6b00825.

[ref20] Kang M. J.; Yu H. J.; Jegal J.; Kim H. S.; Cha H. G. Depolymerization of PET into terephthalic acid in neutral media catalyzed by the ZSM-5 acidic catalyst. Chem. Eng. J. 2020, 398, 125655 10.1016/j.cej.2020.125655.

[ref21] Jie X.; Li W.; Slocombe D.; Gao Y.; Banerjee I.; Gonzalez-Cortes S.; Yao B.; AlMegren H.; Alshihri S.; Dilworth J.; Thomas J.; Xiao T.; Edwards P. Microwave-initiated catalytic deconstruction of plastic waste into hydrogen and high-value carbons. Nat. Catal. 2020, 3, 902–912. 10.1038/s41929-020-00518-5.

[ref22] Marbaix J.; Mille N.; Lacroix L.-M.; Asensio J. M.; Fazzini P.-F.; Soulantica K.; Carrey J.; Chaudret B. Tuning the Composition of FeCo Nanoparticle Heating Agents for Magnetically Induced Catalysis. ACS Appl. Nano Mater. 2020, 3, 3767–3778. 10.1021/acsanm.0c00444. PMC7386363 32743352

[ref23] Pérez-Camacho M. N.; Abu-Dahrieh J.; Rooney D.; Sun K. Biogas reforming using renewable wind energy and induction heating. Catal. Today 2015, 242, 129–138. 10.1016/j.cattod.2014.06.010.

[ref24] Meffre A.; Mehdaoui B.; Connord V.; Carrey J.; Fazzini P. F.; Lachaize S.; Respaud M.; Chaudret B. Complex Nano-objects Displaying Both Magnetic and Catalytic Properties: A Proof of Concept for Magnetically Induced Heterogeneous Catalysis. Nano Lett. 2015, 15, 3241–3248. 10.1021/acs.nanolett.5b00446. 25867032

[ref25] Wang W.; Tuci G.; Duong-Viet C.; Liu Y.; Rossin A.; Luconi L.; Nhut J.-M.; Nguyen-Dinh L.; Pham-Huu C.; Giambastiani G. Induction Heating: An Enabling Technology for the Heat Management in Catalytic Processes. ACS Catal. 2019, 9, 7921–7935. 10.1021/acscatal.9b02471.

[ref26] García-Aguilar J.; Fernández-García J.; Rebrov E. V.; Lees M. R.; Gao P.; Cazorla-Amorós D.; Berenguer-Murcia Á. Magnetic zeolites: novel nanoreactors through radiofrequency heating. Chem. Commun. 2017, 53, 4262–4265. 10.1039/c7cc01138e. 28361140

[ref27] Vinum M. G.; Almind M. R.; Engbæk J. S.; Vendelbo S. B.; Hansen M. F.; Frandsen C.; Bendix J.; Mortensen P. M. Dual-Function Cobalt–Nickel Nanoparticles Tailored for High-Temperature Induction-Heated Steam Methane Reforming. Angew. Chem. 2018, 130, 10729–10733. 10.1002/ange.201804832. 29923289

[ref28] Abu-Laban M.; Muley P. D.; Hayes D. J.; Boldor D. Ex-situ up-conversion of biomass pyrolysis bio-oil vapors using Pt/Al2O3 nanostructured catalyst synergistically heated with steel balls via induction. Catal. Today 2017, 291, 3–12. 10.1016/j.cattod.2017.01.010.

[ref29] Safavinia B.; Wang Y.; Jiang C.; Roman C.; Darapaneni P.; Larriviere J.; Cullen D. A.; Dooley K. M.; Dorman J. A. Enhancing Ce_x_ Zr_1–x_O_2_ Activity for Methane Dry Reforming Using Subsurface Ni Dopants. ACS Catal. 2020, 10, 4070–4079. 10.1021/acscatal.0c00203.

[ref30] Jiang X.; Wang F.; Cai W.; Zhang X. Trisodium citrate-assisted synthesis of highly water-dispersible and superparamagnetic mesoporous Fe_3_O_4_ hollow microspheres via solvothermal process. J. Alloys Compd. 2015, 636, 34–39. 10.1016/j.jallcom.2015.02.156.

[ref31] Wei J.; Yao H.; Wang Y.; Luo G. Controllable Preparation and Catalytic Performance of Magnetic Fe_3_O_4_@CeO_2_Polysulfone Nanocomposites with Core–Shell Structure. Ind. Eng. Chem. Res. 2018, 57, 15039–15045. 10.1021/acs.iecr.8b03191.

[ref32] Kofke T.; Gorte R. J.; Kokotailo G. T. Stoichiometric Adsorption Complexes in [B]- and [Fe]-ZSM-5 Zeolites. J. Catal. 1989, 116, 252–262. 10.1016/0021-9517(89)90090-0.

[ref33] Gorte R. J. What do we know about the acidity of solid acids?. Catal. Lett. 1999, 62, 1–13. 10.1023/A:1019010013989.

[ref34] Kanazirev V.; Dooley K.; Price G. Thermal Analysis of Adsorbed Propanamines for the Characterization of Ga-MFI Zeolites. J. Catal. 1994, 146, 228–236. 10.1016/0021-9517(94)90026-4.

[ref35] Brandrup J.; Immergut E. H.; Grulke E. A. Polymer Handbook, 4th ed.; John Wiley & Sons, Inc.: New York, 1999; Vol. II/365.

[ref36] Kroenlein K. Thermodynamics Source Database, Thermodynamics Research Center. NIST Chemistry WebBook, NIST Standard Reference Database Number 69; Linstrom P. J.; Mallard W. G., Eds., 2020. https://doi.org/10.18434/T4D303 (retrieved December 31, 2020).

[ref37] Brites C. D. S.; Balabhadra S.; Carlos L. D. Lanthanide-Based Thermometers: At the Cutting-Edge of Luminescence Thermometry. Adv. Opt. Mater. 2019, 7, 1801239 10.1002/adom.201801239.

[ref38] Sharma B. K.; Moser B. R.; Vermillion K. E.; Doll K. M.; Rajagopalan N. Production, characterization and fuel properties of alternative diesel fuel from pyrolysis of waste plastic grocery bags. Fuel Process. Technol. 2014, 122, 79–90. 10.1016/j.fuproc.2014.01.019.

[ref39] Kunwar B.; Moser B. R.; Chandrasekaran S. R.; Rajagopalan N.; Sharma B. K. Catalytic and thermal depolymerization of low value post-consumer high density polyethylene plastic. Energy 2016, 111, 884–892. 10.1016/j.energy.2016.06.024.

[ref40] Price G. L.; Kanazirev V. I.; Dooley K. M. Characterization of [Ga]MFI via thermal analysis. Zeolites 1995, 15, 725–731. 10.1016/0144-2449(95)00044-7.

[ref41] Yeh Y.-H.; Gorte R. J. Study of Zn and Ga Exchange in H-[Fe]ZSM-5 and H-[B]ZSM-5 Zeolites. Ind. Eng. Chem. Res. 2016, 55, 12795–12805. 10.1021/acs.iecr.6b03659.

[ref42] Jiang C.; Akkullu M. R.; Li B.; Davila J. C.; Janik M. J.; Dooley K. M. Rapid screening of ternary rare-earth – Transition metal catalysts for dry reforming of methane and characterization of final structures. J. Catal. 2019, 377, 332–342. 10.1016/j.jcat.2019.07.020.

[ref43] López A.; de Marco I.; Caballero B. M.; Adrados A.; Laresgoiti M. F. Deactivation and regeneration of ZSM-5 zeolite in catalytic pyrolysis of plastic wastes. Waste Manage. 2011, 31, 1852–1858. 10.1016/j.wasman.2011.04.004. 21530221

[ref44] Weisz P. B.; Haag W. O.; Rodewald P. G. Catalytic production of high-grade fuel (gasoline) from biomass compounds by shape-selective catalysis. Science 1979, 206, 57–58. 10.1126/science.206.4414.57. 17812451

[ref45] Zhao R.; Macosko C. W. Polymer-Polymer Mutual Diffusion via Rheology of Coextruded Multilayers. AIChE J. 2007, 53, 978–985. 10.1002/aic.11136.

[ref46] Bachus R.; Kimmich R. Molecular weight and temperature dependence of self-diffusion coefficients in polyethylene and polystyrene melts investigated using a modified n.m.r.field-gradient technique. Polymer 1983, 24, 964–970. 10.1016/0032-3861(83)90146-5.

[ref47] Fleischer G. The chain length dependence of self-diffusion in melts of polyethylene and polystyrene. Colloid Polym. Sci. 1987, 265, 89–95. 10.1007/bf01412750.

[ref48] Zupancic I.; Lahajnar G.; Blinc R.; Reneker D. H.; Vanderhart D. L. NMR Self-Diffusion Study of Polyethylene and Paraffin Melts. J. Polym. Sci., Polym. Phys. 1985, 23, 387–404. 10.1002/pol.1985.180230212.

[ref49] Zhu L.; Chiu F.-C.; Fu Q.; Quirk R. P.; Cheng S. Z. D. Polymer Handbook, 4th ed.; John Wiley & Sons, Inc.: New York, 1999; Vol. 5.

[ref50] Bishop M. T.; Langley K. H.; Karasz F. E. Dynamic Light-Scattering Studies of Polymer Diffusion in Porous Materials: Linear Polystyrene in Porous Glass. Macromolecules 1989, 22, 1220–1231. 10.1021/ma00193a038.

[ref51] Kathawalla I. A.; Anderson J. L.; Lindsey J. S. Hindered Diffusion of Porphyrins and Short-Chain Polystyrene in Small Pores. Macromolecules 1989, 22, 1215–1219. 10.1021/ma00193a037.

[ref52] Smit B.; Maesen T. L. M. Molecular Simulations of Zeolites: Adsorption, Diffusion, and Shape Selectivity. Chem. Rev. 2008, 108, 4125–4184. 10.1021/cr8002642. 18817356

[ref53] Kärger J.; Freude D.; Haase J. Diffusion in Nanoporous Materials: Novel Insights by Combining MAS and PFG NMR. Processes 2018, 6, 147 10.3390/pr6090147.

[ref54] Ramírez E.; Larrayoz M. A.; Recasens F. Intraparticle Diffusion Mechanisms in SC Sunflower Oil Hydrogenation on Pd. AIChE J. 2006, 52, 1539–1553. 10.1002/aic.10753.

[ref55] Blyholder G. Molecular Orbital View of Chemisorbed Carbon Monoxide. J. Phys. Chem. 1964, 68, 2772–2777. 10.1021/j100792a006.

[ref56] Siemer M.; Tomaschun G.; Klüner T.; Christopher P.; Al-Shamery K. Insights into Spectator-Directed Catalysis: CO Adsorption on Amine-Capped Platinum Nanoparticles on Oxide Supports. ACS Appl. Mater. Interfaces 2020, 12, 27765–27776. 10.1021/acsami.0c06086. 32432456

[ref57] Hammer B.; Norskov J. K. Why gold is the noblest of all the metals. Nature 1995, 376, 238 10.1038/376238a0.

[ref58] Brogaard R. Y.; Kømurcu M.; Dyballa M. M.; Botan A.; Van Speybroeck V.; Olsbye U.; De Wispelaere K. Ethene Dimerization on Zeolite-Hosted Ni Ions: Reversible Mobilization of the Active Site. ACS Catal. 2019, 9, 5645–5650. 10.1021/acscatal.9b00721. 31205799 PMC6559053

[ref59] Brogaard R. Y.; Olsbye U. Ethene Oligomerization in Ni-Containing Zeolites: Theoretical Discrimination of Reaction Mechanisms. ACS Catal. 2016, 6, 1205–1214. 10.1021/acscatal.5b01957.

[ref60] Ehrmaier A.; Liu Y.; Peitz S.; Jentys A.; Chin Y.-H. C.; Sanchez-Sanchez M.; Bermejo-Deval R.; Lercher J. Dimerization of Linear Butenes on Zeolite-Supported Ni2+. ACS Catal. 2019, 9, 315–324. 10.1021/acscatal.8b03095.

[ref61] Kumar N.; Mäki-Arvela P.; Yläsalmi T.; Villegas J.; Heikkilä T.; Leino A.-R.; Kordás K.; Salmi T.; Yu Murzin D. Dimerization of 1-butene in liquid phase reaction: Influence of structure, pore size and acidity of Beta zeolite and MCM-41 mesoporous material. Microporous Mesoporous Mater. 2012, 147, 127–134. 10.1016/j.micromeso.2011.05.032.

[ref62] Ravi M.; Sushkevich V. L.; van Bokhoven J. A. Towards a better understanding of Lewis acidic aluminium in zeolites. Nat. Mater. 2020, 19, 1047–1056. 10.1038/s41563-020-0751-3. 32958864

[ref63] Dufaud V.; Basset J.-M. Catalytic Hydrogenolysis at Low Temperature and Pressure of Polyethylene and Polypropylene to Diesels or Lower Alkanes by a Zirconium Hydride Supported on Silica-Alumina: A Step Toward Polyolefin Degradation by the Microscopic Reverse of Ziegler–Natta Polymerization. Angew. Chem., Int. Ed. 1998, 37, 806–810. 10.1002/(sici)1521-3773(19980403)37:6<806::aid-anie806>3.0.co;2-6. 29711396

