# Supplementary material for: Catalytic Depolymerization of Waste Polyolefins by Induction Heating: Selective Alkane/Alkene Production
Source: Ind Eng Chem Res. 2021 Oct 14;60(42):15141–50. doi: 10.1021/acs.iecr.1c02674 (PMC8554762; doi:10.1021/acs.iecr.1c02674)
Supplement: Supplementary file 1 — ie1c02674_si_001.pdf [file ie1c02674_si_001.pdf]

Supporting Information

Catalytic Depolymerization of Waste Polyolefins by Induction Heating: Selective Alkane/Alkene Production

Bernard Whajah[1], Natalia da Silva Moura[1], Justin Blanchard[1], Scott Wicker[2], Karleigh Gandar[3], James A. Dorman[1], and Kerry M. Dooley[1]

[1]Cain Department of Chemical Engineering, Louisiana State University, Baton Rouge, Louisiana 70803, United States

[2]Department of Chemistry, Rhodes College, Memphis, Tennessee 38112, United states

[3]Science Department, Baton Rouge Community College, Baton Rouge, Louisiana 70806, United States

The rates (mmol gcat^{-1} s^{-1}) were computed by Eq. 1, the total heat of reaction (J/g) per polymer weight (PE_{wt}) by Eq. 2. Both are reported in Table 1.

$$\frac{\Delta PE_{wt}}{PE_{MW}(CAT_{wt}*\Delta t)}\left(\frac{1000}{60}\right) = Reaction\ Rate \qquad \textbf{Eq. S1}$$

$$\frac{\int H\,dt}{PE_{wt}}(60) = total\ heat/wt \qquad \textbf{Eq. S2}$$

Table S1. PL temperature calibration under N_2 gas.

Temp (°C)	Normalized PL (a.u.)
25	1.000
50	0.937
75	0.897
100	0.847
125	0.800
150	0.738
175	0.672
200	0.603
225	0.540
250	0.459
275	0.388
300	0.332
325	0.273
350	0.238
375	0.204
400	0.183
425	0.164

Table S2. RF temperature response based on normalized PL intensity.

Field Strength	Normalized PL	Temperature
(mT)	(a.u.)	(°C)
0	1.00	23.3
5.4	0.98	32.6
10.8	0.96	43.2
16.2	0.92	62.3
21.6	0.80	122
27	0.52	261
32.4	0.47	285
37.8	0.43	306
43.2	0.36	340
48.6	0.35	346
54	0.29	375
59.4	0.24	399
64.8	0.20	420

Table S3. LDPE conversions for different catalysts with varying applied fields

Catalyst	Amps	Conversion to gas and liquid Products, %	% Aromatics Carbon Basis	Conversion to coke, %	Mol% H_2 / Gas conversion
Fe-Ni-ZSM-5	32	~0	N/A	N/A	N/A
	43	~0	N/A	N/A	N/A
	64	77	0.81	2.0	0.21
Fe-Ni2-ZSM-5	64	58	3.7	2.0	2.1
[1]Fe-Ni2-ZSM-5	0 (350° C)	1.1	0	2.5	6.4
[1]Fe-Ni2-ZSM-5	0 (450° C)	6.5	4.0	3.2	19.0
Fe-Pt-K-MFI	64	82	0.43	0.33	0.02
Fe-Ce-CS-Ni	64	38	4.2	0.56	1.2
(repeat)	64	43	4.8	N/A	N/A
Fe-Ni-Ce-Zr	32	~0	N/A	N/A	N/A
	64	51	5.1	1.1	18
Fe-Ni20-CZA40	64	62	1.9	5.2	33
Fe-Ni	64	49	0.0	7.1	N/A
Fe	64	45	1.2	3.2	1.4

[1]Conventional heating

From the fractional amounts (y) of the high temperature peaks in the TPOs, the coke conversions in Tables 2 and S3 could be determined as follows:

$$\%\ coke\ conversion = \frac{y[W_c+(1-x)W_p]}{W_p} \qquad \textbf{Eq. S3}$$

Where x is the conversion to gas and liquid products, W_c the weight of catalyst, and W_p the initial polymer weight.

The rate constant for use in Eq. 2 was determined from the conversion data in Table 2 for the Fe-Pt-K-MFI catalyst. For a 1st order reaction:

$$-ln(1-f) = \frac{k\,C_0}{N_o}\,V_c\,t = k\,\varepsilon_c\,t \qquad \textbf{Eq. S4}$$

Where f is fractional conversion, C_o the initial concentration of polymer, N_o the initial mols polymer, V_c is the catalyst volume, and ε_c the catalyst/polymer volume ratio. The rate constant has units [fluid vol/(cat vol x time)], or s^{-1}.

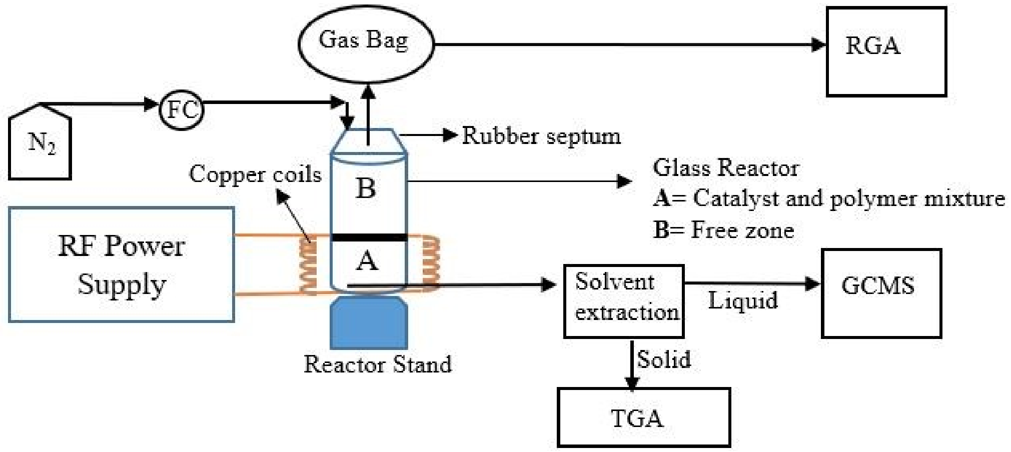

Figure S1. Schematic of the reactor system and product characterization.

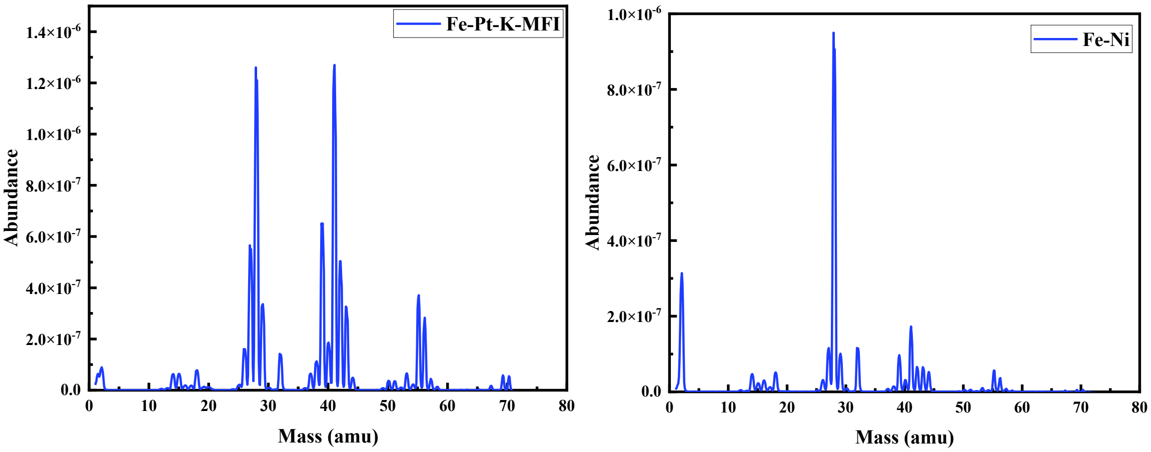

Figure S2. Gas chromatogram by the RGA for the gas produced from RF-induced catalyzed depolymerizations in a 64 mT RF field for 2 h using the Fe-Pt-K-MFI and the Fe-Ni catalysts (115 mg cat: 1000 mg LDPE).

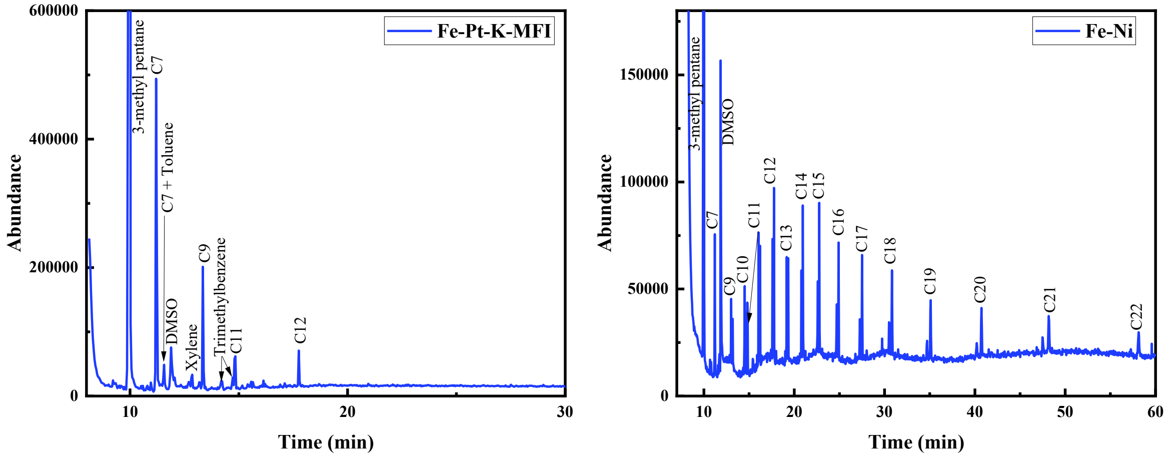

Figure S3. Liquid product GC-MS analysis for RF-induced catalyzed depolymerizations in a 64 mT RF field for 2 h for the Fe-Pt-K-MFI and the Fe-Ni catalysts (115 mg cat:1000 mg LDPE).

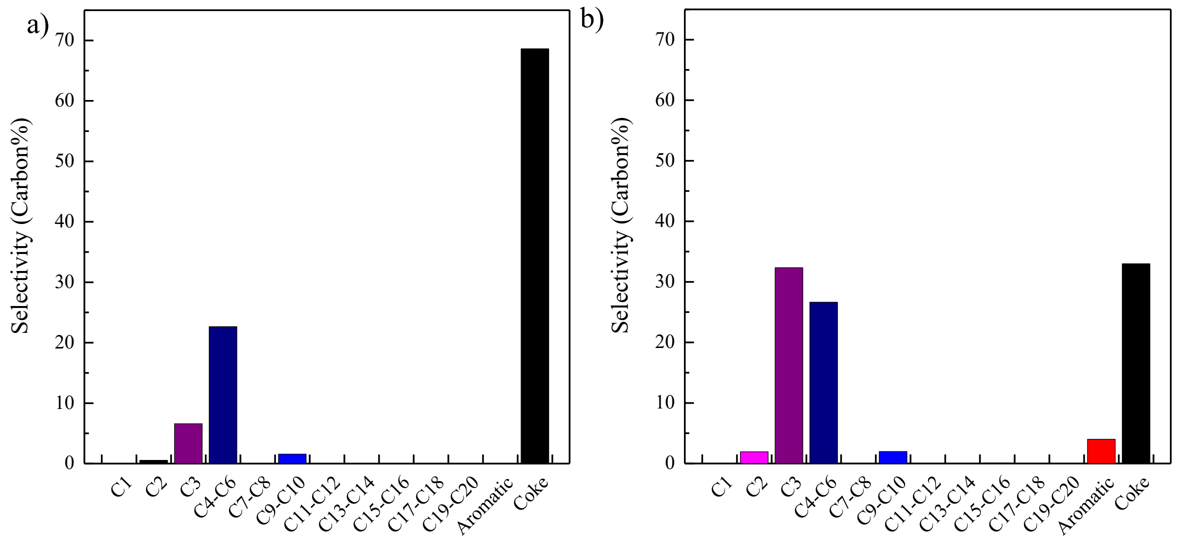

Figure S4. Thermal depolymerization product selectivities on a Carbon% basis run at a) 350 °C and b) 450 °C.

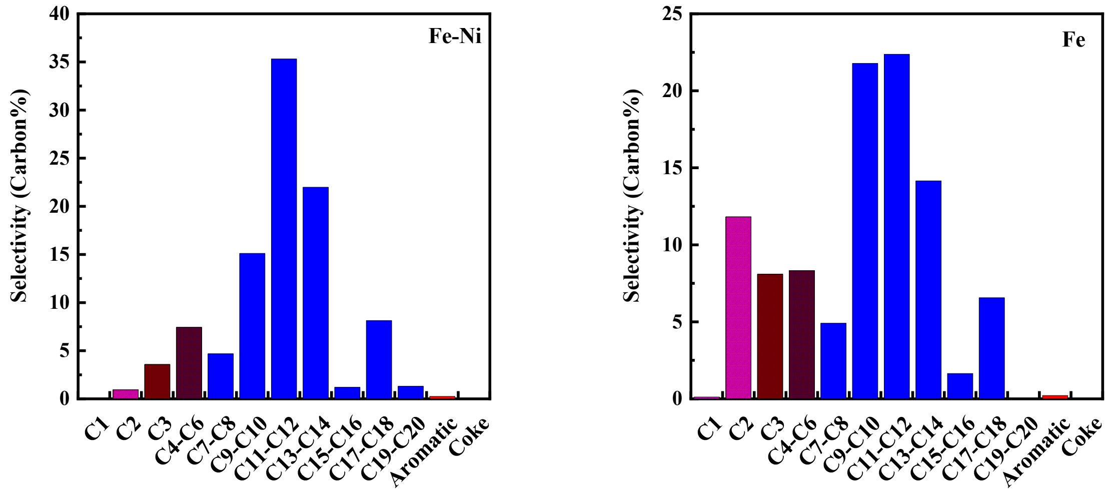

Figure S5. Product selectivities for LDPE depolymerization over: left) Fe-Ni and right) Fe cats at a 200:1000 mg cat:polymer ratio under a 64 mT RF field for 2 h.
